# MiRNA Expression Profile for the Human Gastric Antrum Region Using Ultra-Deep Sequencing

**DOI:** 10.1371/journal.pone.0092300

**Published:** 2014-03-19

**Authors:** Fabiano Cordeiro Moreira, Monica Assumpção, Igor G. Hamoy, Sylvain Darnet, Rommel Burbano, André Khayat, André Nicolau Gonçalves, Dayse O. Alencar, Aline Cruz, Leandro Magalhães, Wilson Araújo Jr., Artur Silva, Sidney Santos, Samia Demachki, Paulo Assumpção, Ândrea Ribeiro-dos-Santos

**Affiliations:** 1 Instituto de Ciências Biológicas, Universidade Federal do Pará, Belém, PA, Brasil; 2 Universidade Federal Rural da Amazônia, Capanema, PA, Brasil; 3 Núcleo de Pesquisa em Oncologia, Universidade Federal do Pará, Belém, PA, Brasil; 4 Centro Regional de Hemoterapia, Faculdade Medicina Ribeirão Preto, Universidade de São Paulo, Ribeirão Preto, SP, Brasil; 5 Instituto de Ciências da Saúde, Universidade Federal do Pará, Belém, PA, Brasil; 6 Hospital Universitário João de Barros Barreto, Universidade Federal do Pará, Belém, PA, Brasil; 7 Secretaria Estadual de Saúde do Estado do Pará, Belém, PA, Brasil; The University of Texas MD Anderson Cancer Center, United States of America

## Abstract

**Background:**

MicroRNAs are small non-coding nucleotide sequences that regulate gene expression. These structures are fundamental to several biological processes, including cell proliferation, development, differentiation and apoptosis. Identifying the expression profile of microRNAs in healthy human gastric antrum mucosa may help elucidate the miRNA regulatory mechanisms of the human stomach.

**Methodology/Principal Findings:**

A small RNA library of stomach antrum tissue was sequenced using high-throughput SOLiD sequencing technology. The total read count for the gastric mucosa antrum region was greater than 618,000. After filtering and aligning using with MirBase, 148 mature miRNAs were identified in the gastric antrum tissue, totaling 3,181 quality reads; 63.5% (2,021) of the reads were concentrated in the eight most highly expressed miRNAs (*hsa-mir-145, hsa-mir-29a, hsa-mir-29c, hsa-mir-21, hsa-mir-451a, hsa-mir-192, hsa-mir-191 and hsa-mir-148a*). RT-PCR validated the expression profiles of seven of these highly expressed miRNAs and confirmed the sequencing results obtained using the SOLiD platform.

**Conclusions/Significance:**

In comparison with other tissues, the antrum’s expression profile was unique with respect to the most highly expressed miRNAs, suggesting that this expression profile is specific to stomach antrum tissue. The current study provides a starting point for a more comprehensive understanding of the role of miRNAs in the regulation of the molecular processes of the human stomach.

## Introduction

MicroRNAs (miRNAs) are small non-coding nucleotide sequences between 17 and 25 nucleotides in length that primarily function in the regulation of gene expression [1], [2]. Studies have demonstrated that miRNAs form a complex, regulatory cell signaling network [2], [3] that results in differentiated gene expression. It is estimated that two-thirds of the human genome is regulated by these small nucleotide sequences. The mechanisms underlying the negative regulation of gene expression by miRNAs are similar in animals and plants, which implies that they are involved in fundamental cellular processes including cell proliferation, development, differentiation and apoptosis [4].

Altered miRNA expression levels may contribute to disease development in humans. Several reports have linked miRNAs to cancer; the first miRNAs to be characterized were involved in cellular proliferation and death. Human tumors and tumor cell lines exhibit large differences in miRNA expression levels compared with normal tissues [5], [6], [7]. The evidence suggests that differentiated miRNA expression may regulate tumor suppressor genes and oncogenes [8].

MiRNA expression profiles have also been studied in many healthy tissues, such as the heart, lung, liver, kidney, brain and cardia mucosa of the stomach [9], [10], [11]. The goal of these types of studies is to establish reference values for future comparisons with altered tissues; thus, expression levels in healthy tissues must be validated. Herein, we proposed to sequence the miRnome of the healthy human gastric antrum region using ultra-deep sequencing.

Epidemiologic changes have been observed in genetic diseases that affect both regions of the stomach, indicating molecular changes in the gastric cardia and gastric antrum mucosa [12]. Identifying the miRNA expression profile in the antrum mucosa and comparing these results with previous studies may elucidate the regulatory mechanisms present in different tissues or regions that are associated with several diseases, including cancer.

## Materials and Methods

### Biological Materials

Our fresh tissue sample for ultra-deep sequencing was obtained from a gastroscopic biopsy (∼4 mm^3^). The patient was 33 years old with no indication of cancer. A macroscopic observation of the tissue showed no evidence of lesions, and a histological examination confirmed the presence of normal and healthy conditions. The sample was collected from a patient at Hospital Universitário João de Barros Barreto/Universidade Federal do Pará in Brazil.

Immediately after the biopsy, a fragment was selected for the experiment. The sample was frozen at –170°C and stored separately in liquid nitrogen in accordance with standard procedure until miRNA extraction.

### Ethics Statement

The ethical guidelines of the Helsinki declaration were followed, and written informed consent was obtained from the patient. This study was approved by the Research Ethics Committee (Comitê de Ética em Pesquisa - CEP) of João de Barros Barreto Teaching Hospital (Hospital Universitário João Barros Barreto - HUJBB) - Federal University of Pará (UFPA) (Protocol number 14052004/HUJBB).

### miRNA Library

The mirVana Isolation Kit (Ambion Inc., USA) was used for total small RNA collection from the tissue sample. The concentration and quality were determined using a Nanodrop 1000 spectrophotometer (ND-1000; Nanodrop Technologies, Wilmington, DE), and purification and size selection were performed using 6% polyacrylamide gel electrophoresis. Using the SOLiD Small RNA Expression Kit (Ambion Inc., USA), 200 ng of small RNA (150–200 bp) was used as a template for the miRNA library. The miRNAs in the library were tagged with unique and specific amplification primers (“the barcode system”; Life Technologies, CA, USA). Next, 50 pg of the library was pooled with seven additional miRNA libraries at the same concentration. A fraction of the library pool (0.1 pg) was amplified and fixed on magnetic beads using emulsion PCR. The ePCR product was deposited onto a single slide, and multiplex SOLiD sequencing was performed.

### SOLiD Ultra-Deep Sequencing and Data Analysis

First, the SOLiD (version 3.0) sequencing system (Life Technologies) was used to generate 35 bp long reads. The second step was to decode the barcode and match each bead sequence to the sample identity. The small RNA sequences data from the gastric tissue are available at European Nucleotide Archive under accession number ERP004687. Sequence analysis was performed using the SOLiD System Small RNA Analysis Tool (Life Technologies) and MiRanalyzer [13]. First, we filtered out the sequences that matched RNA contaminants, such as tRNA, rRNA, DNA repeats and adaptor molecules. After excluding the contaminant reads, we aligned the sequences against miRNA precursor sequences using MirBase (version 19) [14], and we included the reads that matched mature miRNA sequences. To compare the expression data with additional human tissues, miRNA expression data were imported from the microRNA.org database, and the expression for each mature miRNA was normalized by the total read count. A graphical analysis was performed using GenePattern 3.6.1 (http://genepattern.broadinstitute.org).

### miRNA Real-Time PCR (Validation)

The RT-PCR was performed in 10 samples, including the same sample used in Ultra-Deep Sequence. Biopsy samples from gastric antrum tissues were collected from healthy patients and histological examination confirmed the presence of normal and healthy conditions. After collection, the samples were immediately processed and stored at –80°C until RNA extraction. The total RNA was extracted by homogenizing 40 mg of frozen tissue, and the RNA was isolated using the TRIzol reagent (Life Technologies) in accordance with the manufacturer’s instructions. The miRNA concentrations and quality were determined using a Nanodrop 1000 spectrophotometer. The total RNA was reverse transcribed using a TaqMan@- MicroRNA Reverse Transcription kit (Life Technologies).

The miRNA levels were analyzed using a 7500 Real-Time PCR System (Life Technologies) with TaqMan miRNA assays in accordance with the manufacturer’s instructions (Life Technologies); the primers were designed using Primer Express (Life Technologies). The mean expression level for the endogenous Z30 controls was used as an internal control for the miRNA experiments to compare the expression results.

The high miRNA expression levels demonstrated by ultra-deep sequencing (in descending order of expression level*: hsa-mir-145, hsa-mir-29a, hsa-mir-29c, hsa-mir-21, hsa-mir-451a, hsa-mir-192 and hsa-mir-148a*) were validated using TaqMan miRNA assays (Life Technologies).

The following analyses were used to measure the expression levels of mature miRNAs and the inter-individual variation in the 10 samples from healthy antrum tissue. The expression data for each mature miRNA were normalized to the mean expression level in the Z30 human endogenous control. The relative miRNA expression levels were then calculated using the comparative threshold cycle (Ct) method (2^−Δct^).

## Results

Sequencing was performed using the SOLID platform (Life Technologies, CA, USA). The total read count for the antrum tissue exceeded 618,000. After filtering for sequence quality (a minimum of QV≥10 in the first 10 bases) [13] and aligning with MirBase (version 19) [14], 148 mature miRNAs in the human gastric antrum were identified, totaling 3,181 reads. From this set, seven highly expressed miRNAs were selected for validation by RT-PCR.

**Figure 1:**
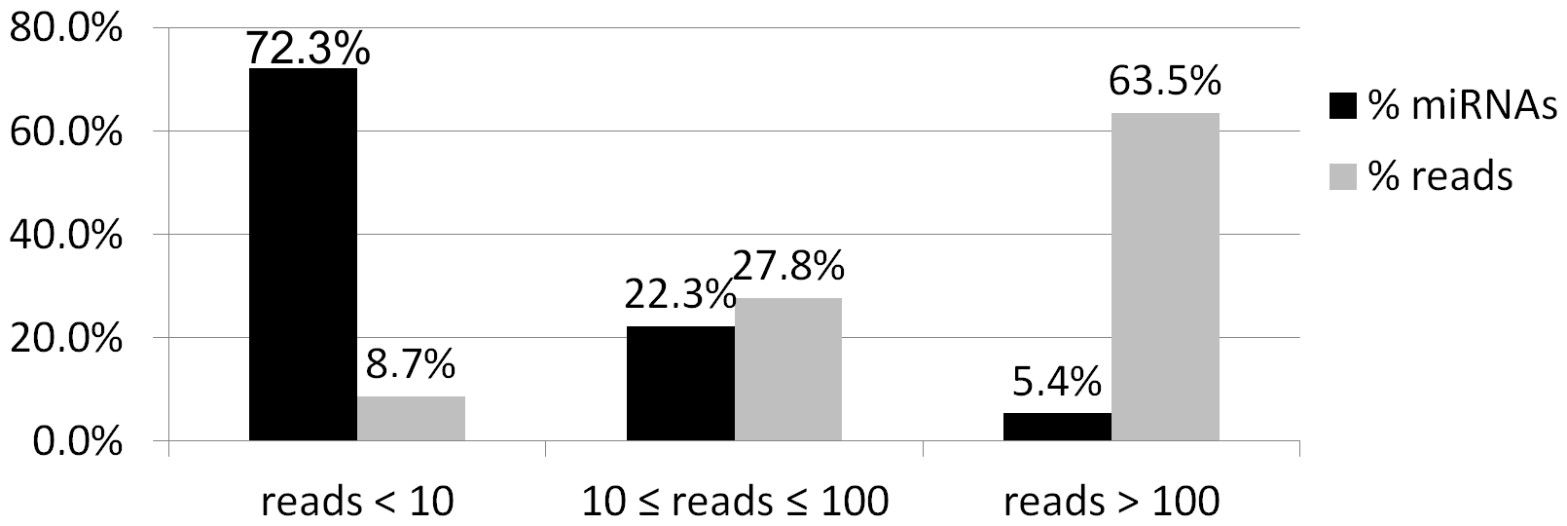
MiRNA distribution in the human gastric antrum by read count number. The miRNA library was divided into three read-count thresholds: i) greater than 100 reads, ii) between 10 and 100 reads and iii) less than 10 reads. Dark gray column: mature miRNA detected by deep-sequencing, grouped by read count. Light gray column: percentage of the respective group in the total count of reads.

For the expression analyses, the miRNA library was divided into three read-count thresholds: i) greater than 100 reads, ii) between 10 and 100 reads and iii) less than 10 reads. A total of 107 miRNAs (72.3%) were expressed at below 10 reads, 33 miRNAs (22.3%) were expressed at between 10 and 100 reads, and 8 miRNAs (5.4%) were expressed at greater than 100 reads. The eight most highly expressed miRNAs (with expression levels greater than 100 reads) produced 2,021 reads, which corresponded to 63.5% of the human gastric antrum tissue total reads (Figure 1). It is worth noting that a small number of miRNAs is responsible for over 60% of the regulation in this region.

**Figure 2:**
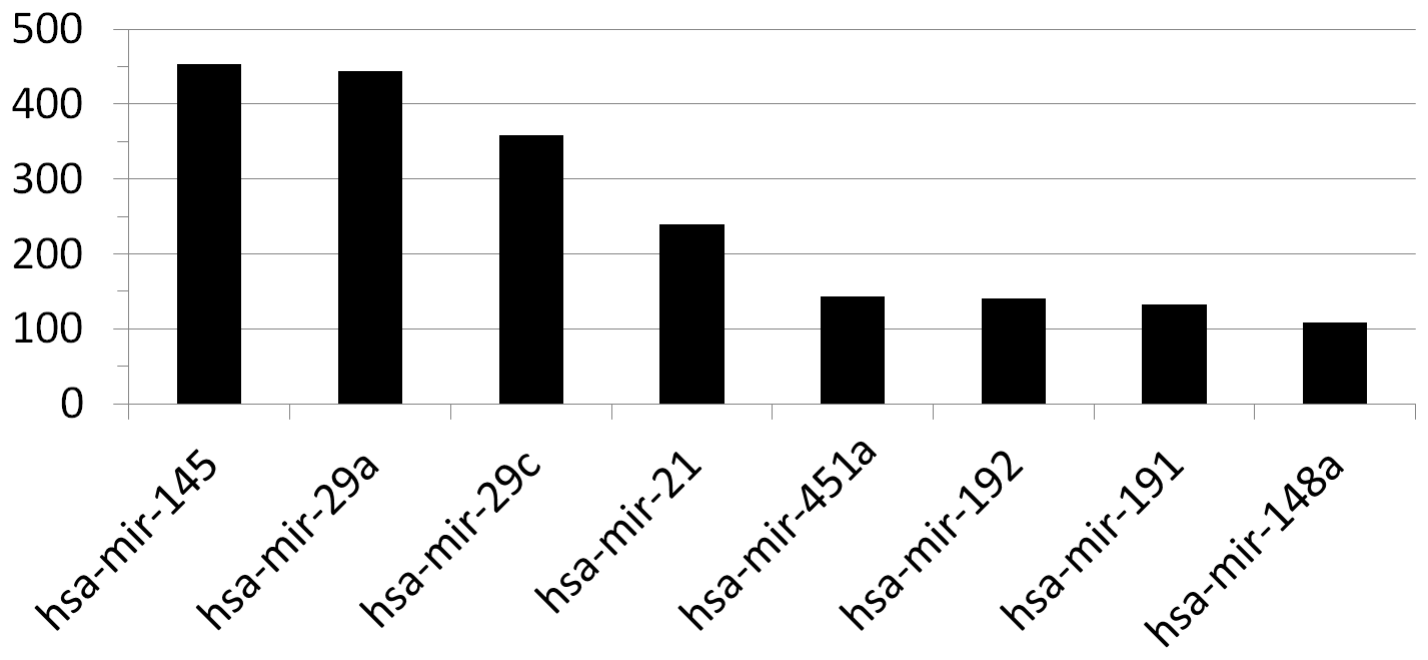
The most highly expressed miRNAs in human gastric antrum region mucosa. The reads counts are based on the number of reads detected during the deep-sequencing of the gastric antrum small RNA library using the SOLiD system. MiRNAs were detected using miRBase (version 19.0).

To characterize the human gastric antrum miRnome profile, the miRNAs with the highest expression levels were selected. Detailed descriptions of these miRNAs are shown in Table 1 and Figure 2. When these results were compared with the cardia’s miRnome [11], it was observed that the miRNAs listed here are among the 15 most highly expressed miRNAs in the cardia, with the exception of *hsa-mir-191*. The number of predicted targets for each miRNA described in Table 1 was obtained from www.microrna.org [15].

**Table 1:** Characteristics of the eight most highly expressed miRNAs in human gastric antrum tissue and the number of possible targets.

miRNA	Read count	Targets number
hsa-mir-145	453	7,464
hsa-mir-29a	444	6,777
hsa-mir-29c	359	6,856
hsa-mir-21	240	5,203
hsa-mir-451a	143	1,605
hsa-mir-192	140	6,545
hsa-mir-191	133	2,015
hsa-mir-148a	109	7,402

**Figure 3:**
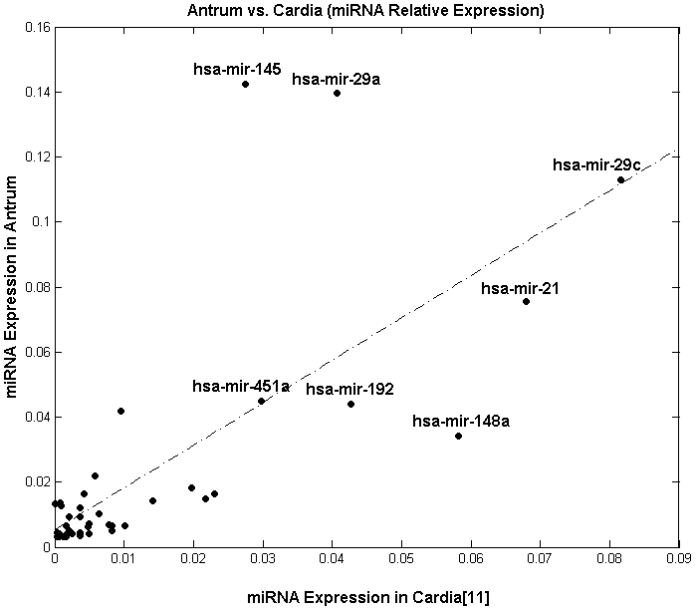
Correlation between the relative expression profiles of the antrum and cardia [11]. For this analysis, all of the miRNAs with at least 10 reads in the antrum were used. The Pearson’s correlation value indicates that the expression profiles of the antrum and cardia tissues are significantly correlated (r = 0.73; p-value < 0.001).

**Figure 4:**
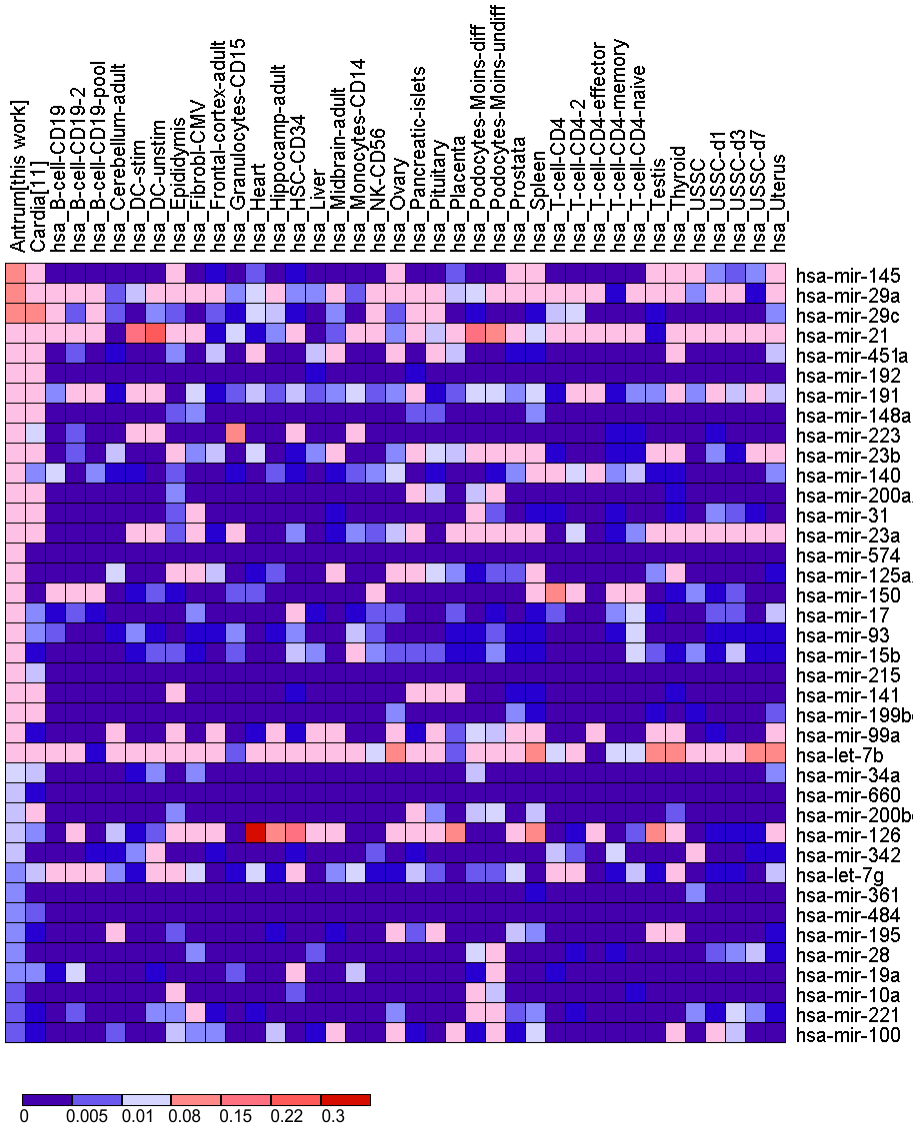
Heatmap of the normalized expression of the most highly expressed mature miRNAs in human gastric tissue in comparison with other normal tissues published in the mammalian microRNA expression atlas [10].

To compare the expression profiles of the antrum and cardia [11] tissues, Pearson’s correlation was applied to all of the miRNAs with at least 10 reads in the antrum (Figure 3). The results indicate that the relative expression levels of the antrum and cardia miRNAs are significantly correlated (r = 0.73; p-value < 0.001). A heatmap was used to compare the expression profiles of both tissues with other known healthy tissue profiles [10] (Figure 4).

The sequencing results for seven highly expressed miRNAs (*hsa-mir-145, hsa-mir-29a, hsa-mir-29c, hsa-mir-21, hsa-mir-451a, hsa-mir-192 and hsa-mir-148a*) were validated using singleplex real-time PCR (qRT-PCR) to determine their expression levels in the gastric antrum region of 10 healthy individuals.

**Figure 5:**
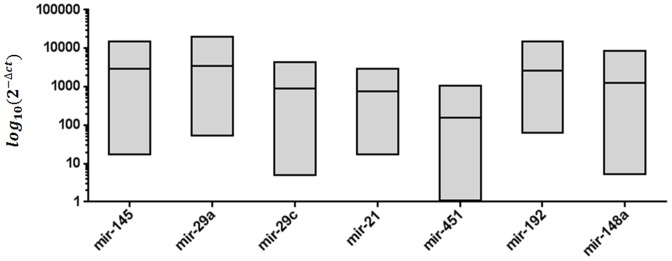
Quantification of the highly expressed miRNAs in the human gastric antrum region mucosa using RT-PCR. Quantification was based on Ct values and normalized using an endogenous Z30 expression control. The 2^(−Δct)^ for each miRNA is the mean of ten replicates of gastric antrum tissue from ten different individuals.

RT-PCR analysis confirmed that all seven of the miRNAs that were identified as highly expressed by the SOLiD platform were also significantly upregulated when compared with the expression levels of endogenous Z30 (p-value < 0.001; Figure 5). Therefore, these seven miRNAs were considered to be over-expressed [7], [16], [17], [18] in the human gastric antrum region.

## Discussion

The total number of miRNA reads found in the present study was 3,181. A total of 63.5% (2,021) of these reads corresponded to the eight most highly expressed miRNAs in antrum tissue. These results suggest the specificity of epigenetic control, where a small number of miRNAs contributes to the majority of the gene regulation. Likewise, in cardia, eight miRNAs are responsible for 57.1% of the gene regulation [11].

Comparing the sequencing results obtained in this study with the cardia miRnome, it was found that seven of the eight most highly expressed miRNAs in the antrum are also among the 15 most highly expressed miRNAs in the cardia [11]. Of the seven miRNAs that were simultaneously expressed in both tissues, five miRNAs maintained a relatively similar expression level after normalization: *hsa-mir-29c, hsa-mir-21, hsa-mir-451a, hsa-mir-192* and *hsa-mir-148a* (Table 2). A more in-depth investigation comparing the miRNAs with at least 10 reads in antrum tissue (Figure 3) indicated that the expression profiles of the cardia and antrum tissues were significantly correlated (r = 0.73; p-value < 0.001). This suggests the presence of an “organ signature” in addition to a tissue signature.

**Table 2:** Read counts of the eight most highly expressed miRNAs in human gastric antrum compared with their relative expression levels in the gastric cardia.

miRNA	% reads Antrum	%reads Cardia
**hsa-mir-145**	14.2	3.6
**hsa-mir-29a**	14.0	5.3
**hsa-mir-29c**	11.3	10.5
**hsa-mir-21**	7.5	8.8
**hsa-mir-451a**	4.5	3.9
**hsa-mir-192**	4.4	5.5
**hsa-mir-191**	4.2	-
**hsa-mir-148a**	3.4	7.5

Moreover, the miRNAs *hsa-mir-145* and *hsa-mir-29a* exhibited significantly different expression levels in the referred tissues, and *hsa-mir-191* was not identified as one of the 15 most highly expressed miRNAs in cardia tissue [11].

The results obtained in this study were compared with the expression profile of additional healthy tissues such as heart, lung, liver, kidney, brain and stomach’s cardia region (Figure 4). Although high expression levels had been previously observed for some of the selected miRNAs in the comparison, the other tissues did not produce a similar miRNA expression profile compared with the profile described herein, which suggests that there is a specific expression profile for the antrum mucosa.

The expression of *hsa-mir-145* and *hsa-mir-29c* has been studied in other tissues, and both miRNAs are known to be cancer suppressor candidates. Their expression levels were found to be upregulated in several healthy tissues. *Hsa-mir-145* is highly expressed in heart, lung, and breast tissues [19], [20], [21], and *hsa-mir-29c* is highly expressed in the stomach and liver [22], [23]. *Hsa-mir-192* is upregulated in gastrointestinal organs and kidney [9]. Although *hsa-mir-21* upregulation has been associated with several diseases, including cancer, it was also found to have high levels of expression in several healthy tissues [10], [11]. In healthy antrum tissue, *hsa-mir-21* was highly expressed, as shown in this study.

Table 2 shows the number of predicted target genes [15] for each of the eight upregulated miRNAs in antrum tissue. An analysis of the potential targets for each miRNA shows that many of the predicted mRNA targets are associated with multiple highly expressed miRNAs in antrum.

A simultaneous analysis of miRNA targets showed that 4,748 different genes may be regulated by up to two of the eight most highly expressed miRNAs, and some genes can be regulated by up to seven of the eight miRNAs. Using the simultaneous presence of at least six miRNAs as a selection criterion, 43 potential target genes were grouped together, as shown in Table 3. For these analyses, only conserved miRNAs with high miSRV scores were used [15], [24].

**Table 3:** Common target genes for at least six of the eight most highly expressed miRNAs in the human gastric antrum region.

miRNAs	HNRNPF	SNRPN	BRWD1	RASSF8	LPP	NCAM1	LAMP2	STRADB	UHRF2	FAM123B	FGD4	PRRG1	TAPT1	NIPBL	ZEB2	HLA-DQB1	PAFAH1B1	PCDH15	SLC25A37	DISC1	MMAA	TET2
mir-145	*	*	*	*	*	*	*	*	*		*	*	*		*			*	*	*	*	*
mir-29a	*	*	*	*		*	*	*	*	*	*	*	*	*	*	*	*	*	*	*	*	*
mir-29c	*	*	*	*	*	*	*	*	*	*	*	*	*	*	*	*	*	*	*	*	*	*
mir-21	*	*	*	*	*	*	*	*	*	*	*	*	*	*		*	*	*	*	*	*	*
mir-451a			*		*																	
mir-192	*	*	*	*	*	*		*		*	*	*	*	*	*	*	*	*	*	*	*	*
mir-191					*		*		*	*				*	*	*	*		*			
mir-148a	*	*		*	*	*	*	*	*	*	*	*	*	*	*	*	*	*		*	*	*

miRNAs	GRIA3	OPRM1	ZFPM2	FBXO28	THAP6	PHF20L1	HIATL1	EML6	SLC39A9	PHTF2	PIGG	ZNF169	GLDN	RPP30	MBTD1	LOC221442	GINS1	ANGEL2	TLE1	CTTNBP2NL	DCUN1D4
mir-145	*	*	*	*	*	*	*	*	*	*	*	*	*	*	*	*	*	*	*	*	
mir-29a	*	*	*	*	*	*	*	*	*	*	*	*	*	*	*	*	*	*	*	*	*
mir-29c	*	*	*	*	*	*	*	*	*	*	*	*	*	*	*	*	*	*	*	*	*
mir-21	*	*	*	*	*	*	*	*	*	*	*		*		*	*	*	*	*		*
mir-451a						*	*		*					*						*	*
mir-192		*			*	*	*	*		*		*	*	*	*	*	*	*		*	*
mir-191	*		*	*	*			*	*		*	*	*		*				*		
mir-148a	*	*	*	*	*	*	*	*	*	*	*	*		*		*	*	*	*	*	*

Only conserved miRNAs with high miSRV scores were used for these analyses [15], [21].

The results suggest that these genes are strong candidates for silencing in the gastric antrum region. Experimental validation of these genes and the functional analysis of each gene may demonstrate a physiological role for these miRNAs in normal gastric tissue.

Several cellular functions are regulated by the miRNA targets selected in this study, including the cell cycle, apoptosis, gene regulation, cell motility and cell migration, which indicates that these genes may play a role in tumorigenesis or tumor suppression. The *LPP* (Lipoma Preferred Partner) gene is target for seven of the eight upregulated miRNAs in the antrum and has been found to be deregulated in several benign and malignant tumors [25]. The *RASSF8, UHRF2, FAM123B* and *STRADB* genes are possible targets for six of the eight most highly expressed miRNAs and are tumor suppressor candidates [26], [27], [28], [29], [30], [31], [32]. *BRWD1* may be regulated by six of the selected miRNAs and is involved in a variety of cellular functions [33].

The *LAMP2, NCAM1, PCDH15* and *LPP* genes are associated with membrane formation and repair, as well as cell-cell adhesion [25], [34], [35], [36]. *NCAM1* may also play a role in the healing processes of gastric ulcers [37]. *HNRNPF, SNRPN* and *BRWD1* are involved in gene regulation and splicing processes [33], [38],[39]. These genes are possible targets for six of the eight most highly expressed miRNAs in antrum tissue.

The *FGD4* and *TAPT1* genes encode proteins that may play roles in cytoskeletal and cell-shape formation [40], [41]. *FGD4* is also involved in mediating cellular invasion by *Cryptosporidium Parvum*, an intracellular parasite that infects the gastrointestinal tract [40]. The *FGD4* and *TAPT1* genes are also targets for six of the selected miRNAs.

This study validates the miRNA expression profile of the stomach’s antrum region tissue and provides complementary information on the role of miRNAs in the molecular regulation processes of healthy human stomach; this information can be used to establish a reference for future comparisons with the altered miRNA expression profiles observed in gastric tract diseases.
